# Bridging Neurofeedback and Structural Connectivity in Chronic Pain

**DOI:** 10.2196/87420

**Published:** 2026-01-28

**Authors:** Alaeddin Acar, Diaa Yahya, Eray Tekirdaş

**Affiliations:** 1 Department of Neurosurgery, Kulu State Hospital, No 4, 139518 Street, Dinek, Kulu, Konya, 42770, Turkey, 90 5424723723; 2 Department of Neurosurgery, Bucak State Hospital, Burdur, Turkey; 3 Department of Neurosurgery, Malatya Training and Research Hospital, Malatya, Turkey

**Keywords:** brain-computer interface, brain oscillations, chronic pain, electroencephalography, musculoskeletal, neuromodulation

We read with great interest and appreciation the article by Bialostocki et al [1] published in *JMIR Research Protocols*. The authors present an innovative electroencephalography-based neurofeedback (EEG-NF) protocol targeting abnormal activity in the right insula (RIns) and dorsal anterior cingulate cortex (dACC) [1].

The authors’ goal of exploring the functional connectivity between these two key regions is particularly noteworthy. This approach aims to modulate the “salience network,” which is involved in processing the emotional-motivational aspects of pain and includes the RIns and dACC as key nodes [2].

At this point, we would like to offer a constructive contribution by approaching the protocol’s underlying mechanism from a neurosurgical perspective. This functional circuit involving the RIns and dACC is thought to operate, at least in part, on a structural foundation provided by the cingulum bundle [2].

This connection is important because invasive modulation of this circuit is a long-established neurosurgical procedure. Anterior cingulotomy is used as a “last resort” option for treatment-resistant, intractable chronic pain syndromes, especially when the affective component of pain is dominant [3]. This procedure creates ablative lesions in the cingulum bundle, permanently interrupting the circuit that Bialostocki et al [1] aim to noninvasively “downtrain.” It is well documented that anterior cingulotomy reduces the emotional response to pain and the component of suffering, rather than the pain intensity itself [3], which is a close parallel to the known functions of the RIns and dACC.

The EEG-NF protocol presented by Bialostocki et al [1] may therefore be viewed, at least conceptually, as a noninvasive counterpart to this invasive surgical approach. Given this parallel, the inclusion of structural imaging methods, such as diffusion tensor imaging (DTI), could add great value to the protocol. The literature, including a systematic review of DTI in migraine, a chronic pain disorder, has shown that the microstructural integrity of key white matter tracts (eg, reduced fractional anisotropy in thalamic radiations, corpus callosum, cingulum, and association fibers) is frequently altered [4].

This raises two important questions for future studies:

1. Could the pretreatment structural integrity of the cingulum bundle, as measured by DTI, be used as a biomarker to predict a patient’s response to this EEG-NF therapy?
2. Can a successful neurofeedback intervention lead to measurable neuroplastic changes in the cingulum bundle, as measured by DTI? Indeed, a strong relationship between functional and structural connectivity has been reported [5].

In conclusion, we believe that adding structural DTI analysis to the innovative protocol by Bialostocki et al [1] may deepen the understanding of the targeted mechanisms, help bridge the gap between noninvasive neuromodulation and invasive neurosurgery, and aid in optimizing patient selection for larger clinical trials in the future.

## Abbreviations

dACC: dorsal anterior cingulate cortex
DTI: diffusion tensor imaging
EEG-NF: electroencephalography neurofeedback
RIns: right insula
